# Incidental Discovery of a Testicular Plasmacytoma at Initial Presentation of Multiple Myeloma

**DOI:** 10.1155/2013/752921

**Published:** 2013-05-07

**Authors:** Amanda R. Hathaway

**Affiliations:** Medical University of South Carolina, 171 Ashley Avenue, Charleston, SC 29425, USA

## Abstract

Testicular plasmacytomas are a rare phenomenon reported in the literature and they can occur as a solitary plasmacytoma, as a recurrence of multiple myeloma, or concurrently in an active myeloma. We report the case of a 43-year-old man who presented with back pain and was diagnosed with multiple myeloma. A CT scan performed to determine the extent of disease revealed an incidental mass in the testicle. Immunohistochemical staining of the mass revealed monoclonal cytolpasmic IgA in the tumor cells and serum studies showed this same immunoglobulin. Following orchiectomy, radiotherapy to the vertebra, chemotherapy with bortezomib, dexamethasone, and doxorubicin, and an autologous bone marrow transplant, the patient is alive twelve months after diagnosis and is in complete remission.

## 1. Introduction

Multiple myeloma is a neoplastic disease accounting for approximately 10% of all hematologic malignancies [1] that normally presents with widespread marrow involvement and can sometimes initially present in extramedullary forms. Extramedullary plasmacytomas (EMPs) have been reported in a variety of locations, most commonly in the respiratory tract, but rare reports have been made of EMP of the testicles. Most reported cases of testicular plasmacytomas have been in either of two settings: as the site of recurrence of multiple myeloma or as a symptomatic mass in the initial presentation of myeloma. 

We report the case of a 43-year-old man with no prior history of multiple myeloma who presents with classic signs of myeloma. During routine workup, an incidental finding of a nonsymptomatic testicular plasmacytoma was discovered. This case is discussed with a review of the literature.

## 2. Case

**Figure 1:**
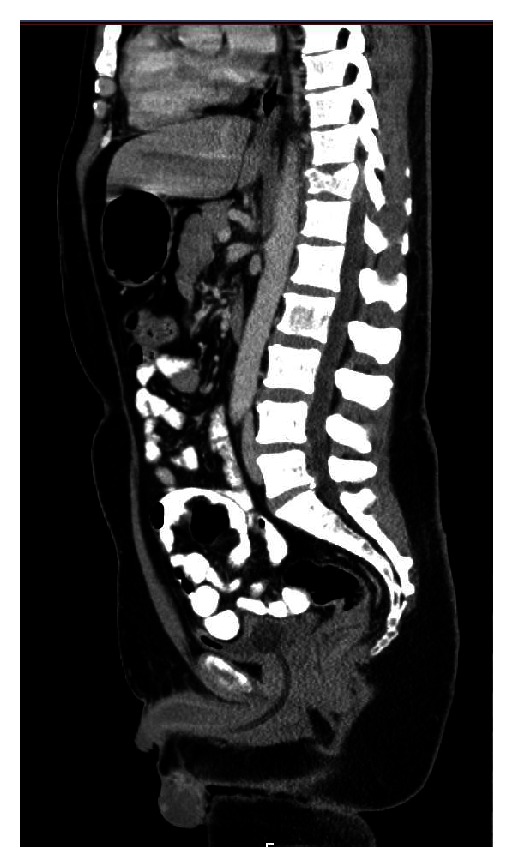
CT scan showing diffuse lytic lesions throughout the axial skeleton.

**Figure 2:**
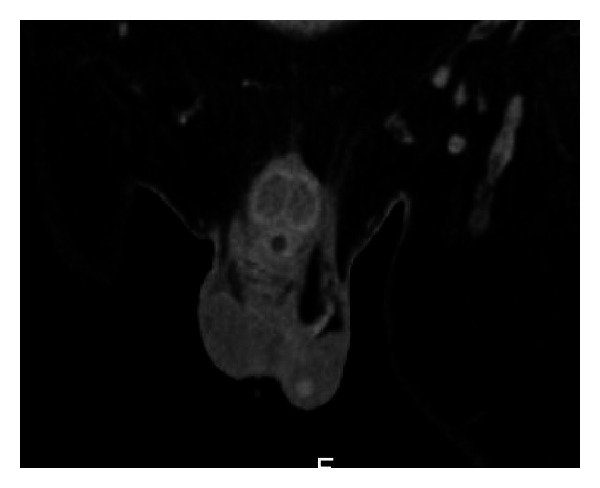
CT scan showing enhancing nodule in the left testicle consistent with malignancy.

**Figure 3:**
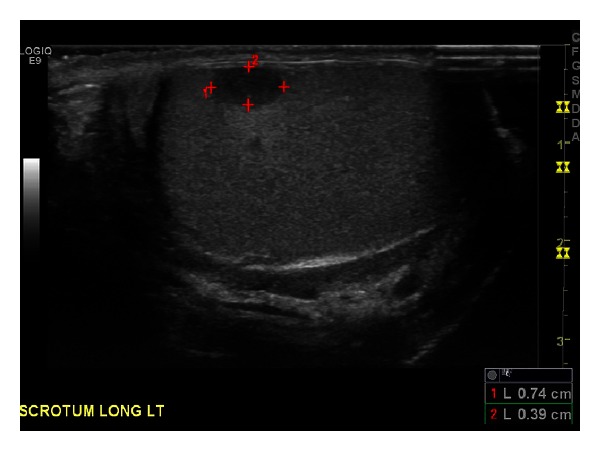
Scrotal ultrasound showed a hypervascular lesion of the left testicle consistent with malignancy.

A 43-year-old African-American man presented complaining of inability to ambulate after a fall attributed to progressive muscle weakness, decreased feeling in his lower extremities, and sensation of “electrical shocks” in his legs for the past two weeks. The patient reported a one-year history of progressive lower back pain that started causing lower extremity weakness over the past two months. He denied a history of fever, fatigue, night sweats, palpable masses or lymph nodes, or weight loss. The patient had no previous significant medical history and, before the back pain began, he lived a normal, active life. Upon examination, the patient had decreased strength to resistance in his bilateral lower extremities, decreased sensation to touch from his toes to the level of his knees, and decreased proprioception and vibration sensation in the toes bilaterally. Reflexes at the knee and ankle were intact. The patient's lungs were clear to auscultation bilaterally and no masses were palpable. Laboratory results were hemoglobin 10.2 g/dL, white blood cells 6.1 × 10^9^/L, platelets 194 × 10^9^/L, creatinine 1.2 mg/dL, calcium 9.7 mg/dL, and total protein 9.8 g/dL. An MRI of the spine showed a compression fracture at T11 with a mass extending into the epidural space at this level, along with a mass at T5-6, and a pleural based mass between ribs T8–T10. The report stated findings were compatible with widespread metastatic disease involving the thoracic spine, lumbar spine, and axial skeleton; a chest-abdomen-pelvis CT was ordered to locate a primary lesion. The CT scan confirmed the presence of diffuse lytic lesions throughout the axial skeleton (Figure 1) and incidentally showed a soft tissue mass from the pleural surface of the posterior medial right lung at T8–T10 and an enhancing nodule in the left testicle consistent with malignancy (Figure 2). A scrotal ultrasound showed a hypervascular lesion of the left testicle consistent with malignancy (Figure 3) and, given the CT findings, the radiologist was concerned for malignant seminoma with diffuse metastatic disease. 

Further laboratory tests were ordered and were as follows: total protein 9.9 g/dL, albumin 3.9 g/dL, ALP 99 U/L, total urine protein 82 mg/dL, ESR 94 mm/h, and LDH 165 IU/L. *β*_2_-microglobulin was 2.75 mg. Serum protein electrophoresis (SPEP) revealed a 2.73 mg/dL IgA lambda monoclonal spike at the beta-2 region with IFE. Urine protein electrophoresis (UPEP) showed an IgA lambda monoclonal spike, and serum free light chains showed a lambda 30 mg/dL, Kappa 0.62, with a ratio of 0.02. The patient was HIV-negative. Serum levels of *α*FP and *β*-HCG were in the normal range. 

The peripheral blood smear showed moderate rouleaux formation of erythrocytes with rare plasma cells (2-3%) present. Bone marrow aspiration showed 35–40% plasma cells and bone marrow biopsy showed sheets of plasma cells of 50–70% with lambda light chain restriction consistent with multiple myeloma. Flow cytometry showed plasma cells with CD38, CD138, CD56 with cytoplasm and lambda light chain restriction consistent with a plasma cell dyscrasia. 

Given the concern for testicular malignancy, the patient underwent unilateral high inguinal orchiectomy. Pathology showed CD138+ and lambda light chain restriction and was consistent with involvement by plasma cell myeloma.

The patient was started on dexamethasone 6 mg IV every six hours and received ten treatments of radiation therapy to the thoracic spine. He was also started on a chemotherapy regimen with bortezomib 1.3 mg/m^2^ days 1, 4, 8, and 11 and dexamethasone 40 mg days 1 through 4. Doxorubicin 4.5 mg/m^2^ was added to cycle two on days 1–4 following completion of radiation therapy. After four rounds of chemotherapy he received an autologous stemcell transplant and on three month follow-up he was in complete remission.

## 3. Discussion

Multiple myeloma is a neoplastic disorder in which there is a malignant production and proliferation of a monoclonal population of plasma cells causing an increased secretion of paraprotein throughout the body. Multiple myeloma accounts for around 1% of all cancers and approximately 10% of all hematologic malignancies [1]. The clinical presentation of multiple myeloma involves lytic bone lesions, hypercalcemia, and renal impairment, due to the increased amount of paraprotein dispersed throughout the body as a result of the spread of neoplastic plasma cells in the bone marrow [2]. The neoplastic plasma cells are thought to mainly propagate in the bone marrow and eventually spread into adjacent bone, but there have been several reports of malignant plasma cells invading other tissues and organs [3]. Rosenberg et al. describe the disease as a “chameleon-like tumor,” as is it able to mimic other normal and pathologic processes and spread into any organ or tissue in the body [4]. The spread of these cells is thought to happen when clonogenic pre-malignant cells circulate in the bone marrow until they find an environment favorable for the differentiation and eventual expansion into myeloma [5].

Extramedullary plasmacytomas (EMPs) are collections of neoplastic plasma cells that occur outside of the bone marrow. In a study of 869 cases of extramedullary plasmacytomas unrelated to myeloma, it was discovered that around 16% of EMPs eventually transform in multiple myeloma, with most transformations happening in the first two years following diagnosis of the EMP [27]. EMPs are common in patients with concurrent multiple myeloma or as a form of recurrence of myeloma in previously treated patients. The incidence of EMPs varies depending on the study. In histological analysis at autopsy of patients with myeloma, Weitzner reported the incidence of EMP to be between 11 and 73% of these cases [6]. In autopsy studies of patients with multiple myeloma, Hayes et al. found microscopic disease in the liver, spleen, and lymph nodes in 70% of these patients, but reported that gross EMPs in other locations were much less common and rarely affected more than one anatomical site [7]. Another study of 62 consecutive autopsies of patients with multiple myeloma determined that 28% of patients had some form of EMP [8]. Overall, it is believed that between 65 and 71% of myeloma patients have some form of EMP [7, 9]. Gross evidence of EMPs is most commonly found in the respiratory and gastrointestinal tracts, but there have been reports of EMPs located in a variety of different organs and soft tissues, with testicular involvement being especially rare. Generally the prognosis of EMP without evidence of multiple myeloma is good; 5-year survival rates reach as high as 70% and median survival time is around 8 years [3, 10]. In general, EMPs outside of the head and neck locations have a poorer prognosis [11]. EMPs in the case of multiple myeloma have a far worse prognosis and the presence of EMP in the testicles is considered evidence of extensive disease.

Testicular involvement by extramedullary plasmacytomas is considered a rare event; only 71 cases documented as of 2008 [3] with the first case documented in 1939 [12]. Hayes et al. performed autopsies on 38 patients with multiple myeloma with EMPs and found evidence of testicular involvement in only one case. They further reviewed the literature going over 182 cases with similar demographics and found only 5 cases of EMP with testicular involvement [7]. Hellwig [13] and Pasmantier and Azar [9] performed 128 and 57 autopsies, respectively, of patients with EMP and neither reported any cases of testicular involvement. Another study of 161 cases of EMP documented only one case of testicular involvement [7]. According to a study review of 6,000 tumors in the American Testicular Tumor Registry, 7 were reported to be plasmacytomas [14]. A review of tumors from the English Testicular Tumor registry reported only 3 of 2,700 testicular tumors as plasmacytomas [15]. These studies report that the overall incidence of testicular EMP in multiple myeloma is between 0.6 and 2.7% and the incidence of testicular plasmacytomas being 0.03–0.1% of all testicular tumors [7, 14, 16]. 

There has been debate concerning whether testicular EMP is a separate entity or if it is part of multiple myeloma, but it is now widely accepted that testicular plasma-cell neoplasia is a local manifestation of a systemic disease process [14, 17–19]. It has been proposed that the testis is a sanctuary site from chemotherapy due to a blood-testis barrier created by the lower temperature in the scrotum [4, 20]. Because of this barrier, several of the documented reports of testicular plasmacytomas have been cases of a plasmacytoma as the first indication of recurrence of multiple myeloma [4, 5, 7, 14, 16, 21–24]. There are fewer reports in the literature of testicular plasmacytomas at initial presentation of multiple myeloma [3, 25, 26]. Anghel et al. reviewed all the reported cases of testicular plasmacytomas from 1939 to 2002 and of the 34 total cases, 14 patients had a previous diagnosis of multiple myeloma, 6 had had a primary EMP, one had bone lytic lesions and EMP, and four had another EMP at presentation of the testicular plasmacytoma [5]. Of the 34 cases, only 10 cases of testicular plasmacytoma showed evidence of simultaneous bone marrow involvement or a diagnosis of multiple myeloma at presentation. 

The data suggest that testicular plasmacytoma with concurrent multiple myelma is a poor prognostic indicator, with overall median survival of four months [5]. Other studies have concluded that overall survival of patients with EMP of the testicle is poor, with overall survival following orchiectomy to be between 5 weeks and 48 months [21] and with 59% of patients with testicular plasmacytomas succumbing to the disease between 9 days and 26 months despite orchiectomy, radiotherapy, and chemotherapy [5]. It is estimated that 80% of testicular plasmacytomas eventually have an association with multiple myeloma, with an overall average survival of 12 months [17]. 

Our patient presented with signs and symptoms of multiple myeloma and the incidental finding of a testicular plasmacytoma was discovered. Testicular plasmacytomas can be the first manifestation of multiple myeloma, evidence of recurrence of myeloma, or a rare location of a plasma cell dyscrasia, but most often they show no clinical manifestations and are discovered on autopsy [18]. When symptoms arise from testicular plasmacytomas, they usually present as a testicular mass without pain unless it is complicated by other organ or tissue involvement [3, 5, 27].

Using immunoperoxidase techniques, Avitable et al. determined that testicular tumors produced the same Ig idiotype as the disseminated disease [21]. It was later determined that testicular plasmacytomas have a higher incidence of IgA involvement than classical multiple myeloma. In a review of seven reported cases of solitary testicular plasmacytomas analyzed with immunoperoxidase staining, the majority were the IgA idiotype [5] although other subtypes have been reported. In classical myeloma, around 25% of cases are of the IgA class (Alexanian), but in cases of testicular plasmacytomas, the IgA incidence is 46.7%. Our patient presented with IgA idiotype, conforming to the majority of cases with testicular plasmacytomas. This raises the question of whether the IgA idiotype is more aggressive or if the IgA subtype has a greater predilection towards causing plasmacytomas and is something that could be further examined.
